# Knowledge and Awareness of Cancer Genome Profiling Tests among Japanese Patients with Cancer

**DOI:** 10.3390/clinpract14050166

**Published:** 2024-10-12

**Authors:** Yuko Kawasaki, Tamotsu Sudo, Kazuo Tamura, Saki Hinoshita, Kayoko Hasuoka, Satoko Miyawaki, Nao Matsutani, Akira Hirasawa, Atsuko Uchinuno

**Affiliations:** 1 College of Nursing Art and Science, University of Hyogo, Akashi 673-8588, Japan; 2 Department of Cancer Genomics, Fujita Health University Hospital, Toyoake 47-1192, Japan; 3 Graduate School of Science and Engineering Research, Kindai University, Higashiosaka 577-8502, Japan; 4 Department of Nursing, Hyogo Cancer Center, Akashi 673-8588, Japan; 5 Department of Nursing, Okayama University Hospital, Okayama 700-8558, Japan; 6 Department of Nursing, National Hospital Organization Shikoku Cancer Center, Matsuyama 791-0280, Japan; 7 Cancer Genetics and Genomics Service, National Hospital Organization Kyushu Cancer Center, Fukuoka 811-1395, Japan; 8 Department of Clinical Genomic Medicine, Graduate School of Medicine, Dentistry and Pharmaceutical Sciences, Okayama University, Okayama 700-8558, Japan; 9 School of Nursing, Tsuruga Nursing University, Tsuruga 914-0814, Japan

**Keywords:** cancer, genome profiling, cancer genomic medicine

## Abstract

(1) Background: The number of patients with cancer undergoing cancer genome profiling is increasing; however, it remains unclear how accurately they understand the details of the tests and treatments. This study aimed to clarify the awareness of cancer genome profiling tests among patients with cancer who visited cancer genome medical clinics. (2) Methods: A questionnaire survey was conducted on awareness, anxiety, sources of information, and psychological states concerning cancer genome profiling tests. (3) Results: In total, 265 patients with cancer (117 men, 142 women, 6 no response, average age of 58.29 ± 11.9 years) were included in the study, of which 218 (82.3%) were aware of the term “cancer genomic medicine” and 90 (34.0%) were aware of its details. Thus, only a few respondents understood that cancer genome profiling tests facilitate the discovery of secondary findings and of genes associated with hereditary tumors. Regarding their psychological state when visiting the cancer genome clinic, the respondents were anxious about standard treatment and prognosis limits. (4) Conclusions: From the viewpoint of advance care planning, we suggest that medical professionals build a support system that links palliative care and cancer treatment and coordinates genetic counseling at an early stage.

## 1. Introduction

The recent development of next-generation sequencers capable of reading gene sequences at high speed has enabled the comprehensive detection of a large number of gene mutations that are therapeutic targets in a short period of time. This technology has been established in cancer genomic medicine, making it possible to select drugs that match genetic mutations in a patient’s cancer tissue. In Japan, cancer genomic profiling was included in insurance coverage in June 2019 [1]. Using 48,627 samples from the Center for Cancer Genomics and Advanced Therapeutics (C-CAT), the comparison with White patients in the Genomics Evidence Neoplasia Information Exchange (GENIE) demonstrated high TP53 mutation frequencies in Asian patients across multiple cancer types [2]. The most common cancers in Japan are colorectal, lung, stomach, prostate, and breast cancer [3]. In addition, the number of patients with cancer undergoing cancer genome profiling is increasing; however, it remains unclear how accurately they understand the details of the tests and treatments [4].

Concerns remain about cancer genomic profiling. Currently, 80–90% of the patients tested have yet to undergo an effective treatment beyond standard therapy. Patients with a pathogenic variant or a variant of uncertain significance (VUS) experience increased psychological distress. Similarly, factors such as low education levels, low genetic knowledge scores, and younger age contribute to this distress [5]. Patients with cancer and their families may be aware of the risks associated with somatic cell mutations and genetic information related to germline mutations (hereditary tumor). Health professionals need to consider economic, technical, and medical factors to select the appropriate test for their patients [6].

Regarding the awareness and use of genetic testing in the United States, 75% of participants were aware of genetic testing, and 19% had undergone the procedure [7]. Prior research has studied patient reactions after notification of the test results; however, no study has been conducted on patient perceptions or attitudes prior to testing.

This study aimed to clarify the awareness of cancer genomic profiling tests among patients with cancer who visited cancer genome medical clinics.

## 2. Materials and Methods

Participants were recruited from four representative medical hospitals with outpatient sections specializing in cancer genome testing in Japan. In Japan, cancer genome profiling tests are currently being considered for individuals with (1) solid tumors for which no standard treatment is available or (2) locally advanced or metastatic solid tumors in whom standard treatment has been completed or is nearing completion, and who wish to receive the next available drug therapy. Patients with cancer attending a cancer genome medical outpatient section were included, and those aged <18 years or with difficulty answering the questionnaire were excluded. The sample size was 273 patients with a population ratio of 10, maximum permissible error of 6%, and confidence level of 90%, based on the number of new cancer cases in Japan in 2018 being 980,856.

The study period was from October 2019 to September 2021. A questionnaire survey was used with the following items: (1) basic information: age, sex, location, and stage of cancer; (2) awareness, anxiety, and desired information concerning cancer genome profiling and easy-to-use information; and (3) the psychological state of the patient receiving the cancer genome profiling (nursing record). The level of recognition was measured by selecting from three levels (know, know a little, do not know at all).

Data analysis was performed using SPSS 26.0 (IBM Corp., Armonk, NY, USA) to calculate means, standard deviations, and percentages for variables representing participant characteristics, knowledge, and perceptions of cancer genomic medicine. Independent samples t-tests were used to compare group characteristics. Free-text content was extracted using content analysis methods to identify exploratory behavior, motivations for taking the test, and psychological reactions.

All patients who visited the outpatient clinic during the study period were given an overview of the study. Thereafter, potential participants were provided a written request for participation explaining the purpose and methods of the study, information about the confidentiality of their data, the voluntary nature of participation, stringent protocols for storage and disposal of personal information, and publication of the study results. Only those who agreed to participate completed the questionnaires. Data were collected anonymously using serial IDs, ensuring data confidentiality. This study was conducted in line with the Helsinki declaration and was approved by the ethics committee of the university with which the authors are affiliated (approval number: 2019F09).

## 3. Results

### 3.1. Overview of Respondents

**Figure 1:**
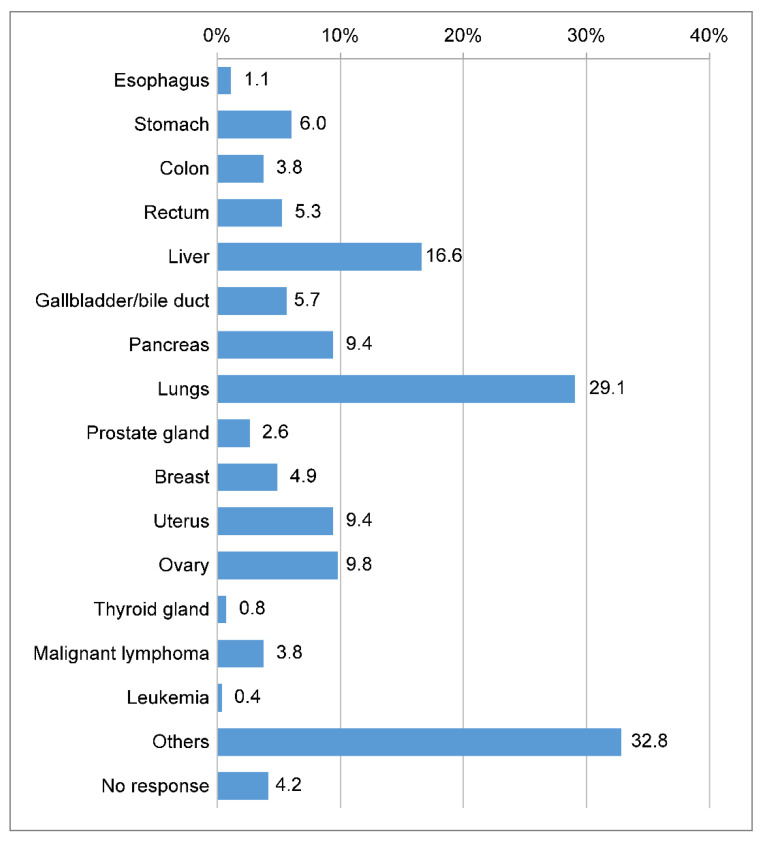
Time of cancer diagnosis. Q: Where is your cancer located? (Please circle where applicable).

In total, there were 117 men and 142 women, with six participants providing no responses. The respondents were an average age of 58.29 ± 11.9 years old. As for when they were diagnosed with cancer, 94 (35.5%) answered “≥1 year but <3 years”, and 54 (20.4%) answered “≥5 years”. Figure 1 shows the location of the cancer; 141 (53.2%) respondents had stage 1, 9 (3.4%) had stage 2, 29 (10.9%) had stage 3, and 141 (53.2%) had stage 4 cancer.

The most prevalent symptom experienced by respondents was “numbness” in 106 respondents (40.0%), followed by “lethargy” in 86 (32.5%), “hair loss” in 76 (28.7%), “loss of appetite” in 64 (24.2%), “anxiety” in 57 (21.5%), and “back pain” in 56 (21.1%).

Chemotherapy was the most common form of treatment in 210 respondents (79.2%), followed by surgery in 51 (19.2%), and radiation therapy in 25 (9.4%). Emotional regulation coping was employed by 136 (51.3%) respondents, problem-focused coping by 99 (37.4%), and avoidance coping by 4 (1.5%) for managing stress. Overall, 191 (72.1%) respondents had family members who had received a cancer diagnosis.

### 3.2. Term Awareness

**Figure 2:**
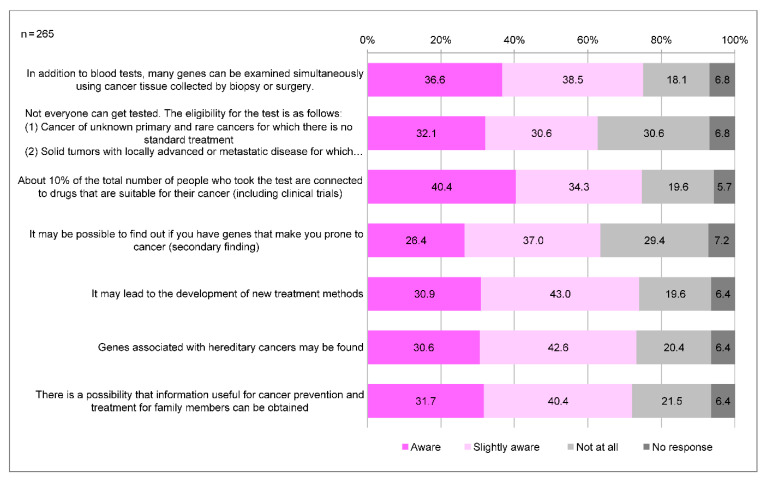
Awareness of the details of cancer genome profiling. Q: Please read each sentence regarding “cancer genome medicine” and circle the applicable section.

**Figure 3:**
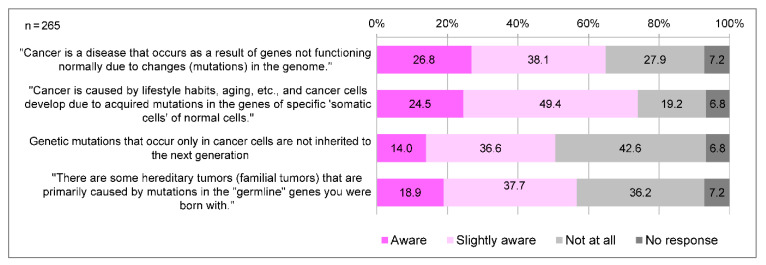
Awareness of the details of cancer and genome/gene. Q: “Cancer, genome/genes” is explained below. Please read each sentence and circle the appropriate section.

In Japan, cancer genome medicine is positioned as a medical treatment that utilizes cancer tissues to simultaneously examine a large number of genes to identify genetic mutations, thereby tailoring treatment and other measures to each individual’s unique genetic profile and disease state. A total of 218 (82.3%) participants were aware of the term “cancer genomic medicine”, and 90 (34.0%) were aware of its details. The awareness of the significance of cancer genome profiling tests is generally low; only a few people knew that cancer genome profiling tests allow for the discovery of secondary findings and of genes associated with hereditary tumors (Figure 2). Regarding genomic changes and cancer development, respondents tended to be less aware of “germline” gene mutations (18.9%), and that “somatic” genetic mutations are not inherited by the next generation (14.0%) compared to “somatic” gene mutations (24.5%) (Figure 3).

### 3.3. Anxiety

**Figure 4:**
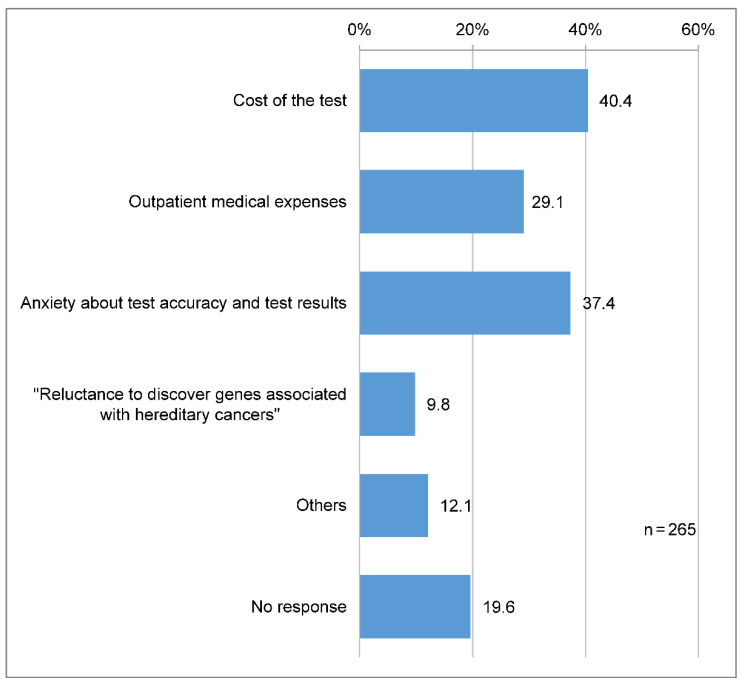
Concerns about undergoing cancer genome profiling. Q: What makes you feel anxious or stressed about receiving “cancer genomic medicine”? (Multiple answers are allowed).

A total of 107 (40.4%) participants reported that when receiving cancer genomic medicine, they were most anxious and stressed about the cost of the test, and 99 (37.4%) about the test accuracy and results (Figure 4).

### 3.4. Information Needs

**Figure 5:**
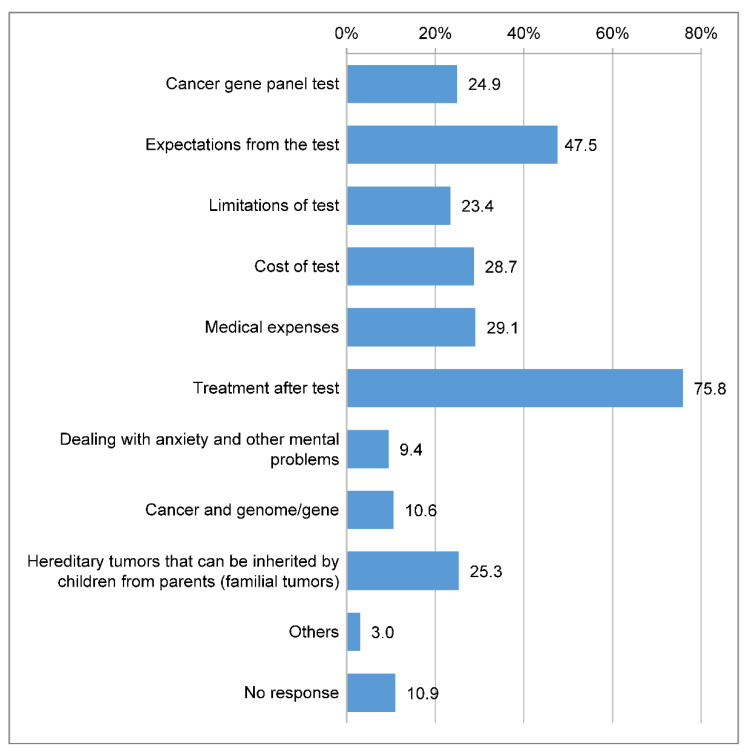
Information respondents want to know about cancer genome profiling. Q23: What information would you like to know about “cancer genomic medicine”? (Multiple answers are allowed).

In total, 201 (75.8%) respondents indicated that they wanted information about treatments after the test, and 126 (47.5%) expressed a desire to understand the expectations from the test, which was the highest among the items related to information needs (Figure 5).

### 3.5. Accessible Resources

**Figure 6:**
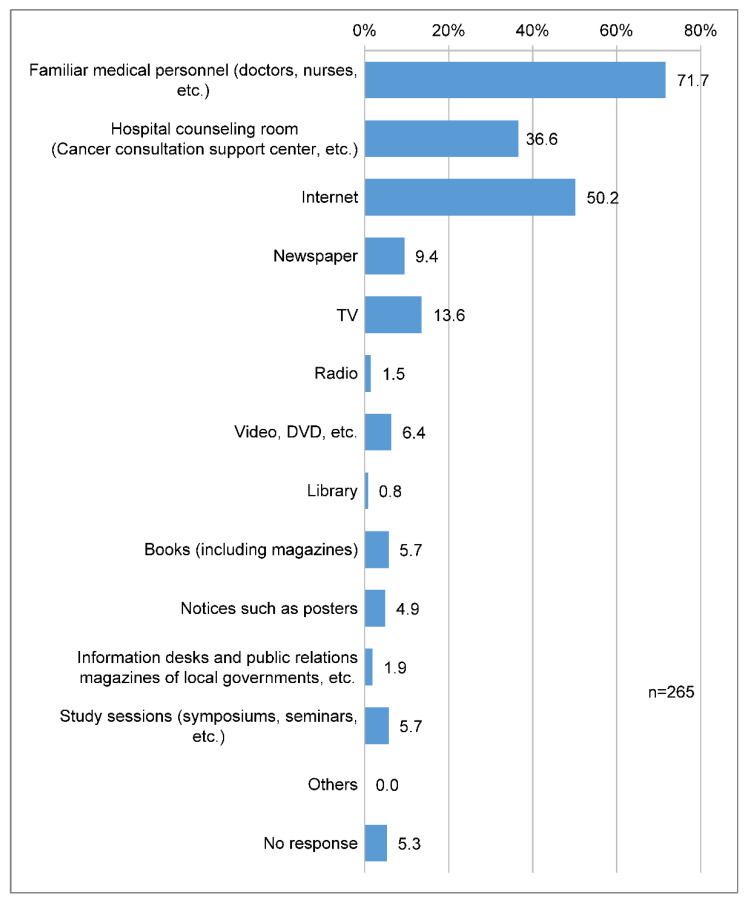
Accessible sources of information about cancer genome profiling. Q: What do you think is the easiest way to obtain information about “cancer genomic medicine”? (Multiple answers are allowed).

Nineteen (71.7%) respondents said that medical professionals were the most accessible means of acquiring information on cancer genomic medicine, followed by the internet (133 respondents, 50.2%) and hospital consultation offices (97 respondents, 36.6%) (Figure 6).

### 3.6. Sharing Test Results with Family Members

In total, 237 (89.4%) people wished to share information with their family members when genes associated with hereditary tumors were found.

### 3.7. Exploratory Behavior, Motives for Taking the Test, and Psychological Reaction before the Test

From the recorded data describing the reactions of patients with cancer and their families, we extracted elements related to exploratory behavior, test-taking motivation, and psychological reactions that encouraged patients with cancer to undergo genome profiling, which revealed the following.

#### 3.7.1. Exploratory Behavior

Patients with cancer search for new treatments, including treatments with less burden and treatments for rare cancers, as they face the limitations of standard cancer treatments.

#### 3.7.2. Motivations for Taking the Test

The motivations for taking the cancer genome profiling test included recommendations from others, expectations for the development of future treatment methods, expectations for maintaining the current lifestyle, and expectations for life extension. Furthermore, the respondents correctly understood the limitations of the test and were willing to undergo it, assuming that receiving it would lead to measures to prevent cancer in their children.

#### 3.7.3. Psychological Reaction

As for the psychological state when visiting the cancer genome clinic, the respondents were anxious about the limits of standard treatment and the limited prognosis. Conversely, the respondents accepted their current disease and tried to have a positive attitude by possessing a positive outlook on their prognosis, undergoing tests to avoid regret, and relying on tests when they were in a predicament.

## 4. Discussion

Since the respondents of this survey were patients who had visited the Cancer Genome Outpatient Clinic, 82.3% of them were familiar with the term “cancer genome medicine”; however, their awareness of the significance of cancer genome profiling tests was generally low. This can be attributed to the fact that in Japan, individuals with solid tumors who have completed or are scheduled to complete standard treatment and who wish to receive the next available drug therapy consider cancer genome profiling testing. Regarding the awareness and use of genetic testing in the United States, participants with a family history of cancer were more likely to be aware of cancer genetic testing than those without, and participants with a personal history of cancer were more likely to have undergone cancer genetic testing [5]. Similarly, in the present study, 72.1% of the respondents had a family member with a cancer diagnosis.

Respondents of this survey expressed concern about the cost of testing and test results when they did not fully understand specific terms, such as “genomics” and “somatic and germline mutations”. As their comprehension of these tests and terminologies improved, their concerns shifted to the potential implications of having a VUS or germline mutation. Regarding the details of the test, we found that respondents had low awareness of hereditary tumors and secondary findings. However, we clarified that many patients with cancer assume their germline gene mutations will be revealed and express a desire to share test results with their family members. This is thought to be influenced by the vague idea that “cancer is hereditary”, which means that respondents did not sufficiently understand hereditary tumors. In this regard, The Impact of Genetic Counseling Educational Tools, a picture book explaining the six words found in prior research (mutation, germline mutation, somatic mutation, biomarker, molecular testing, and targeted therapy), is difficult to understand [8], and The Know Gene Scale of 16 items [9] should be used to develop an essential understanding of hereditary tumors in patients with cancer. Furthermore, the desire to share test results with family members is a significant action that will lead to cancer prevention in the next generation.

Respondents in this survey expressed concerns about having germline mutations and indicated willingness to undergo testing due to their understanding that it could lead to cancer prevention measures for their children. This reflects the perceived significance of the test. In a survey of the attitudes of patients with metastatic breast cancer toward cancer genome profiling, 33% correctly remembered and described their genomic test results. A study reported that 39% of the respondents who remembered participating in a clinical trial experienced decision-making conflicts [10]. Respondents who underwent genetic testing were more likely to rely on their healthcare providers and genetic counselors for decision-making. Respondents who underwent genetic tests also reported less reliability of sources other than doctors: the internet and social media (odds ratio = 0.33; *p* < 0.001) and journals and magazines (odds ratio = 0.48; *p* = 0.007) [11]. Therefore, we believe that it is necessary for medical professionals to support the patients, ensuring that they can accurately understand the test results, make decisions about their treatment options, and share information with family members.

Concerns regarding the test include cost and accuracy, and we have clarified that there are high expectations for future treatments. The most accessible information sources were familiar medical professionals (doctors, nurses, etc.), the internet, and hospital consultation rooms. This indicates that patients are seeking information about the possibility of future treatment rather than being concerned about them. The results of a patient awareness survey for large-panel genomic tumor testing (GTT) showed that patients had high expectations that they would benefit from GTT (M = 2.81 on a 0–4 scale) and positive attitudes toward it (M = 2.98) [12].

Participants had little knowledge about genes or breast cancer risk, but most felt that regarding genetic testing for cancer prevention, “everyone should get the test” (87.7%). Most of the participants had positive opinions regarding genetic testing for cancer prevention [13]. Thus, in general, the opinion is positive regarding genetic testing for cancer prevention. However, some barriers were noted, including genetic testing not being offered in a nearby clinic (46.9%), insurance companies knowing the results (54.0%), cost (49.1%), and no accessible genetic counselors with whom to discuss results (45.6%) [14]. With this in mind, the patient has high expectations regarding the possibility of finding a treatment suitable for their cancer and is likely to undergo the test. However, depending on the test results, there is a possibility that an appropriate treatment may not be found or that a hereditary tumor may be found. Therefore, we believe it is necessary to sufficiently explain in advance the significance of the test and the possibility of treatment based on test results.

Many of the respondents of this survey decided to undergo testing in the period leading up to the test. As they explored treatment possibilities, their expectations and anxieties about the test grew. Regarding psychological reactions, we found that some patients were worried about the prognosis because the treatment they were currently receiving was no longer effective, and they decided to undergo the test because of this predicament. These psychological reactions can be triggered if the patient undergoing the test has advanced cancer. In a survey of patients who underwent gene expression profile (GEP) testing, the patient’s understanding of GEP testing varied, and patients valued the test because it gave them certainty amidst confusion, options, a sense of empowerment, and personalized, authoritative information [15]. Thus, medical professionals need to be aware of the diversity of patients’ understanding of the test and their desire to gain a sense of security by expanding treatment options through testing. In particular, in Japan, cancer genome profiling tests are considered for individuals with solid tumors who have completed or are scheduled to complete standard treatment and who wish to receive the next available drug therapy. Thus, patients who have undergone examinations when standard treatment is no longer effective and are searching for treatment possibilities and anticipating the development of future treatment may be conflicted or anxious about their limited prognosis.

Given this situation, health care providers need to improve communication to ensure that the patients are fully informed about the significance of the test and the possibility of treatment before undergoing the test, allowing patients to share the information with their families. In addition, for individuals with solid tumors who have completed or are scheduled to complete standard treatment and undergo testing to seek the next available drug therapy, healthcare providers should integrate palliative care and cancer treatment as part of advance care planning. They should actively provide information to help cancer patients understand the significance and limitations of the test. When a gene related to a hereditary tumor is found, we propose establishing a support system that facilitates genetic counseling.

This study had some limitations. Firstly, this is a cross-sectional study with a sample size and is limited in explaining causal relationships because of the easy introduction of biased effects. The possibility of selection bias exists because the survey was administered through a questionnaire and the participants were limited to four cancer genome medical clinics. Since the sample of this study included patients with cancer in Japan, its applicability to other countries needs to be examined. Future research is warranted to explore communication methods tailored to the perceptions of patients undergoing cancer genome profiling testing (e.g., disease status, significance of testing).

## Data Availability

The data presented in this study are available on request from the corresponding author due to ethical reasons.
